# Pathophysiological effects of androgens on the female vascular system

**DOI:** 10.1186/s13293-020-00323-6

**Published:** 2020-07-29

**Authors:** Tori Stone, Nina S. Stachenfeld

**Affiliations:** 1 John B. Pierce Laboratory, 290 Congress Ave, New Haven, CT 06510 USA; 2 Department of Obstetrics, Gynecology and Reproductive Sciences, Yale School of Medicine, New Haven, CT USA

**Keywords:** Transgender, Trans men, Testosterone, Endothelial function, Blood pressure

## Abstract

Sex hormones and their respective receptors affect vascular function differently in men and women, so it is reasonable to assume they play a role in the sex differences in cardiovascular disease states. This review focuses on how the effects of testosterone on arterial vessels impact the female vasculature. In women with androgen-excess polycystic ovary syndrome, and in transgender men, testosterone exposure is associated with high blood pressure, endothelial dysfunction, and dyslipidemia. These relationships suggest that androgens may exert pathophysiological effects on the female vasculature, and these effects on the female vasculature appear to be independent from other co-morbidities of cardiovascular disease. There is evidence that the engagement of androgens with androgen receptor induces detrimental outcomes in the female cardiovascular system, thereby representing a potential causative link with sex differences and cardiovascular regulation. Gender affirming hormone therapy is the primary medical intervention sought by transgender people to reduce the characteristics of their natal sex and induce those of their desired sex. Transgender men, and women with androgen-excess polycystic ovary syndrome both represent patient groups that experience chronic hyperandrogenism and thus lifelong exposure to significant medical risk. The study of testosterone effects on the female vasculature is relatively new, and a complex picture has begun to emerge. Long-term research in this area is needed for the development of more consistent models and controlled experimental designs that will provide insights into the impact of endogenous androgen concentrations, testosterone doses for hormone therapy, and specific hormone types on function of the female cardiovascular system.

## Introduction

Most studies on vascular dysfunction have been conducted on male subjects, in part due to a general assumption (in the early part of the twentieth century) that women were at low risk for generalized cardiovascular diseases. Further, it was generally assumed that physiological responses in women would be similar to those of men. Excluding women from research studies resulted in an overall failure to recognize the important and differential effects of reproductive hormones on the female cardiovascular system. In 1993, the US Congress passed the NIH Revitalization Act (https://grants.nih.gov/grants/funding/women_min/guidelines.htm) directing the National Institutes of Health to ensure the inclusion of women in clinical research unless “a clear and compelling rationale and justification establishes to the satisfaction of the relevant Institute/Center Director that inclusion is inappropriate with respect to the health of the subjects or the purpose of the research.” Cardiovascular disease is recognized as the leading cause of mortality in both men and women in the USA [1]. Moreover, the influence of sex hormones in cardiovascular health is complex and related to age, environment, sex differences in the tissues exposed, and, in the case of exogenous hormones, the route of administration. The current review highlights the impact on the female vascular system when exposed to high levels of androgens.

### Androgens

**Fig. 1:**
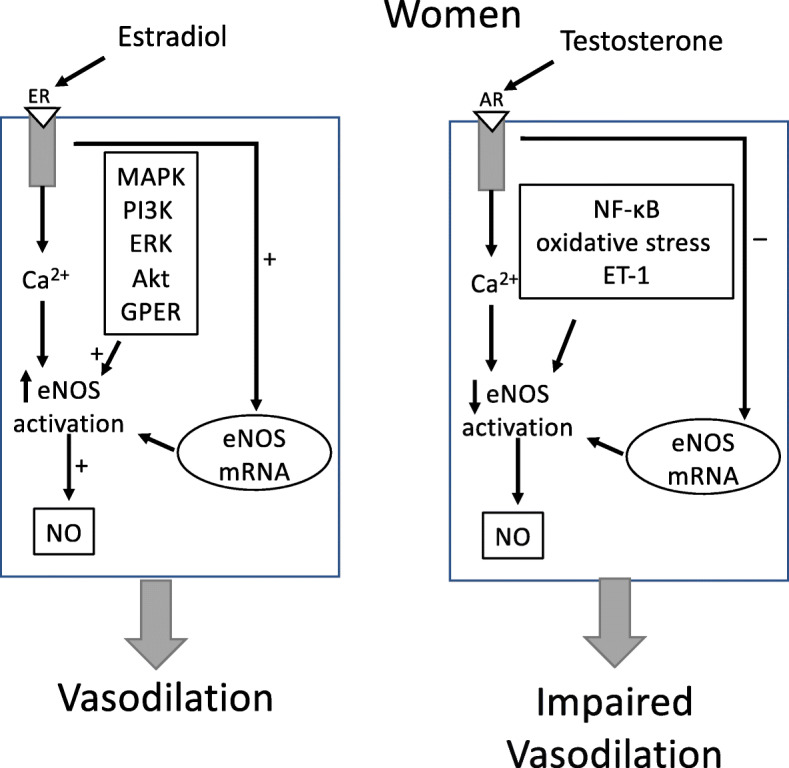
Sex differences in the signal transduction pathways of eNOS activation in endothelial cells by testosterone and the subsequent vascular response. Testosterone is generally thought to increase nitric oxide (NO) availability through genomic and non-genomic pathways in men. Testosterone works through a separate pathway in women resulting in reduced NO availability and impaired vasodilation. Androgen receptor (AR), endothelial NO synthase (eNOS), extracellular signal-regulated kinase (ERK), phosphatidylinositol 3-kinase (PI3K), Akt (protein kinase B), G protein-coupled estrogen receptor 1 (GPR30 agonist, G-1). From Stanewicz et al. [7], with permission

In men, testosterone is synthesized in testicular Leydig cells and secreted by the testes. Approximately 10% of testosterone is converted via 5-alpha reductase to the potent dihydrotestosterone (DHT). This conversion to DHT primarily occurs in target tissues such as prostate, seminal vesicles, and hair follicles, and then binds to the androgen receptor (AR) specific to that tissue. The adrenals in both men and women secrete small amounts of testosterone that is quickly converted to estrogen via aromatase. In women, the ovaries also produce small amounts of testosterone, most of which are quickly aromatized to estrogen. In both men and women, the aromatization of testosterone to estrogen is mainly a peripheral effect taking place in a number of tissues throughout the body, including blood vessels, brain, fat, skin, and bone. Androgens regulate gene transcription when ligand-activated and also may induce rapid activation via kinase signaling cascades and mechanisms independent of transcription [2–5], such as activation of intracellular Ca^2+^ [5], MAPK [3], Akt [2], and PKA/PKC [6] (Fig. 1). Androgen receptors are expressed in cells throughout the vascular system, including endothelial cells [8] and vascular smooth muscle cells [9]. The AR primarily mediates androgenic effects on the endothelium, and some androgen effects on the vascular system are mediated indirectly through estrogen [10] after testosterone conversion by aromatase [8]. Androgen actions on the endothelium are often mediated by NO [11–13]. These actions vary significantly in men because both estradiol exposure and aromatase levels fluctuate widely.

### Androgens in females

Estrogens may play a cardioprotective role at certain stages of life in females [14–16], meaning that women with high estrogen exposure have a lower incidence of cardiovascular disease or coronary artery disease (CAD) compared to age-matched men [15]. Androgens appear to induce unfavorable responses in the female vascular system [17, 18]. Engagement of androgens and the AR leads to impaired agonist-triggered endothelial NO release in women [19]. For example, high circulating androgen levels in women, such as in androgen-excess PCOS (AE-PCOS), is associated with high circulating inflammatory cytokines, oxidative stress, and NF-κB activation [20], all of which impair NO release and lead to endothelial dysfunction [21] and mild hypertension [22]. Androgen exposure is associated with endothelial dysfunction in women, including elevated ET-1 levels, independent of insulin resistance, obesity, or fertility status [19]. Some [23–25], but not all [26, 27] studies suggest that as women age and enter perimenopause, when both androgens and estrogens are fluctuating [28], androgens are more pronounced and are associated with coronary artery calcification and carotid intima-media thickness.

The sex differences in vascular function are well recognized and impact clinical presentation, pathophysiology, treatment, and response to treatment [29]. Sex hormones affect vascular function differently in men and women and, therefore, likely contribute to these differences in vascular function [7, 30, 31]. There is evidence that the engagement of androgens and the AR represent a causative link between sex differences and vascular function and induce detrimental outcomes on the female vascular system [31–36]. These effects are in contrast to men, in whom androgen effects can be positive or negative depending on the physiological environment and factors that disrupt it [5]. Androgens can increase reactive oxygen species and decrease NO bioavailability in females, so likely contribute to increases in blood pressure [37].

### Androgen-excess polycystic ovary syndrome

Polycystic ovary syndrome (PCOS), the most common reproductive endocrinopathy affecting ~ 1 in 10 women, is the most common cause of infertility in women. Approximately, 75% of women with PCOS have the more severe endocrine and metabolic PCOS phenotype, AE-PCOS, that is dominated by clinical and biochemical manifestations of hyperandrogenism. The pathophysiology involves dysregulation of a number of endocrine signals, including altered pulsatility of hypothalamic gonadotropin-releasing hormone (GnRH) from the arcuate nucleus, resulting in altered secretion of the pituitary gonadotropins luteinizing hormone (LH) and follicle-stimulating hormone (FSH). This disordered hypothalamic function is associated with excess production of androgens under the influence of both elevated LH and excess insulin as well as disordered insulin action in target tissues and failure of ovarian folliculogenesis due to the altered gonadotropin signaling and hyperandrogenemic milieu. Ovaries of AE-PCOS women are replete with oocytes containing immature follicles, but mechanisms that underlie the dynamics of normal follicular growth and egg maturation are disordered. Thus the “cysts” of AE-PCOS ovaries represent arrested follicles that contain immature eggs. Finally, in addition to androgen excess, AE-PCOS is a state of progesterone deficiency resulting from chronic failure to achieve ovulation.

**Fig. 2:**
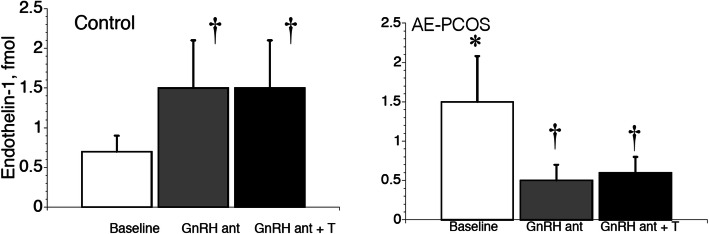
Plasma endothelin-1 (P_[ET-1]_) concentration increases in control women but decreases in women with androgen excess-polycystic ovary syndrome (AE-PCOS) during testosterone (T) suppression with a gonadotropin releasing hormone antagonist (GnRH ant). Asterisk indicates greater baseline P_[ET-1]_ in AE-PCOS compared to control. Dagger indicates changes in P_[ET-1]_ during T suppression and T administration, *P* < 0.05. Data from Wenner et al. [40], with permission

The overexposure to androgens is considered the key disruption influencing the clinical features of AE-PCOS. The mechanisms responsible for vascular dysfunction in AE-PCOS are currently unknown, but research also points to the chronic excess androgen milieu [38–41]. The androgenic effects on cardiovascular risk are already apparent in young women with AE-PCOS [20, 21, 42–44] and characterized by endothelial dysfunction [45–48]. Endothelial dysfunction is an early sign of atherosclerosis, hypertension, and diabetes and usually driven by impaired agonist-triggered endothelial NO release [49]. Impairments in agonist-triggered endothelial NO release are evident in women with AE-PCOS and exacerbated by increased circulating inflammatory cytokines, oxidative stress, and NF-κB activation [20] that contribute to the endothelial dysfunction [21] and result in mild hypertension [22]. Thus, chronic hypertension and endothelial dysfunction [21] are prevalent in young women with AE-PCOS [50]. The altered vascular function is concomitant with a spectrum of other covert risk markers of CAD, including obesity, insulin resistance, an atherogenic lipid profile, and proinflammatory milieu [50]. Endothelin-1 is one of several circulating indicators of endothelial injury [51, 52] and poor endothelial function [53]. In our studies in women with AE-PCOS, we demonstrated elevated ET-1 compared to control obese, insulin-resistant women. We also demonstrated that short-term (4–7 days) of androgen suppression with a GnRH antagonist reduced ET-1 (Fig. 2) [21, 54]. Finally, administering methyltestosterone while continuing the GnRH antagonist had little impact on ET-1 levels in either group (Fig. 2) [40]. From our results in women with AE-PCOS, we concluded that androgens drove dysfunction in the ET-1 system and the associated poor endothelial dysfunction. From these same studies, we also showed an increase in ET-1 during GnRH antagonist administration in our control obese, insulin-resistant group without AE-PCOS, likely due to estrogen suppression (Fig. 2) [40].

**Fig. 3:**
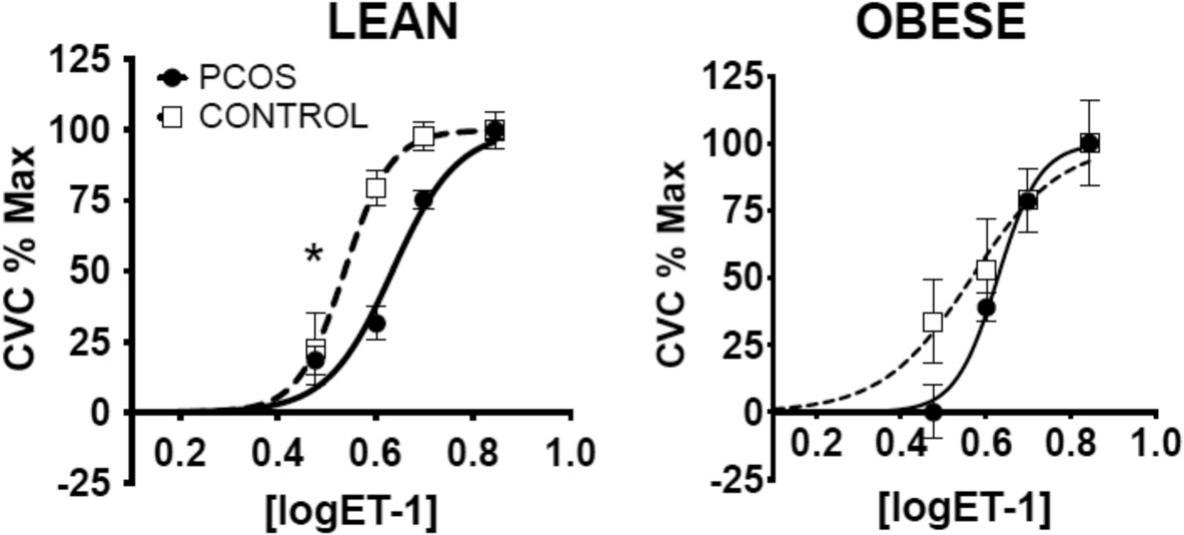
Dose-response curves during cutaneous microdialysis perfusions of low dose endothelin-1 (ET-1) in lean (left) and obese (right) women with androgen excess polycystic ovary syndrome (AE-PCOS) and control subjects. Percent maximal cutaneous vasodilation (CVC %Max). Asterisk indicates difference from Control within lean BMI group. Data are presented as means ± SEM. From Usselman et al. [38], with permission

Although the prominent phenotype of young women with AE-PCOS manifests a spectrum of covert CAD risk markers (including obesity, insulin resistance, hypertension, an atherogenic lipid profile, and a proinflammatory milieu), we hypothesized that their poor endothelial mediated vascular function is driven by elevated androgen exposure. Recent data from our laboratory supported this hypothesis when we demonstrated poor vascular function in lean women with AE-PCOS (Fig. 3) [38]. In these studies, endothelial function was compromised in lean, insulin-sensitive women with AE-PCOS indicating the relation of AE-PCOS to vascular dysfunction is independent of insulin resistance, obesity, and fertility status [19], and supporting our hypothesis that endothelial dysfunction in AE-PCOS was a consequence of elevated androgen exposure. These data provided strong evidence that elevated androgen levels have a negative impact on endothelial function and overall vascular function in women with AE-PCOS.

We have also studied the impact of testosterone effects on the autonomic control of blood pressure in women with AE-PCOS. The arterial baroreflex is a key homeostatic mechanism that regulates fluctuations in blood pressure that occur with each heartbeat. Women with AE-PCOS show poor baroreflex function [55]. Further, AE-PCOS is also associated with impaired cardiac (cardiovagal) baroreflex [56], which contributes to sympathovagal dysfunction during standing [57] and impaired autonomic control [58]. In general, plasma testosterone levels and peripheral, i.e., muscle sympathetic neural activity (MSNA), are positively correlated [59]. Women with AE-PCOS also demonstrate increased spontaneous MSNA [60] and resting blood pressure versus healthy controls, as well as decreased sympathetic baroreflex gain—i.e., the slope of the relationship between MSNA and blood pressure, further demonstrating impaired autonomic control of blood pressure in women with AE-PCOS [55]. It follows that elevated testosterone in women may impair sympathetic baroreflex control of arterial blood pressure. In support of this hypothesis, the testosterone-MSNA relationship is more powerful in lean compared to obese women with AE-PCOS [60, 61]. These findings also indicate that testosterone is an independent predictor of MSNA in AE-PCOS [60, 61].

### Transgender men

While studies on AE-PCOS have successfully demonstrated the effects of chronic androgen exposure on endothelial function and blood pressure regulation in women exposed to chronically high androgens, there are many confounding cardiovascular disease risk factors associated with PCOS. In contrast, young, healthy transgender men receive levels of testosterone (40-100 mg/week) to achieve male physiologic levels (400-1000 ng/mL testosterone) during gender-affirming hormone therapy (HT) to achieve female-to-male gender transition, and doses are adjusted to address the patients’ goals and responses [48, 62]. Increasing testosterone exposure creates extremely high androgen exposure to the female cardiovascular system. Thus, research on the impact of these male physiologic doses of androgens on female cardiovascular and other systems is needed and provides an opportunity to isolate the impact of testosterone on vascular function in the female cardiovascular system.

Approximately 1.4 million people in the USA (0.6% of the population) identify as transgender [63] or having a current gender identity that differs from the sex assigned at birth. Cisgender individuals have a gender identity the same as the sex assigned at birth. Transgender men are persons assigned female at birth with a male gender identity. Gender affirming hormone therapy to reduce characteristics of their natal sex and induce those of their desired sex is the primary medical intervention sought by transgender people. While HT is recognized by the World Professional Association for Transgender Health as medically necessary [64], there are significant medical risks associated with HT [65], including potential cardiovascular risk [66–68]. Reduced ovarian hormones concomitant with increased testosterone during HT is associated with increased systolic blood pressure [48], dyslipidemia [48, 66], endothelial dysfunction [48], and thrombosis risk markers in young trans men [69]. Further, attention to the androgen effects on the CV system in trans men is especially important because HT continues throughout their lifetime into old age.

Many transgender individuals begin HT at an early age when the overall risk for cardiovascular events is still low. Therefore, it is important during HT to evaluate biomarkers and risk factors that predict cardiovascular disease later in life. Importantly, even in these younger trans men cohorts, HT is associated with increased systolic blood pressure in some [70–72] but not all studies [73, 74], and is also associated with increased triglycerides (TG) [72, 74], LDL-cholesterol and decreased HDL-cholesterol [66, 71]. A recent meta-analysis revealed that in a female-to-male transgender population, testosterone administration increased serum TG and LDL-cholesterol and decreased HDL-cholesterol at 3, 6, and 24 months [66]. Cardiovascular morbidity is not yet apparent in these young trans men [67], and these studies did not follow subjects to an age when risks of CAD are known to accelerate.

**Fig. 4:**
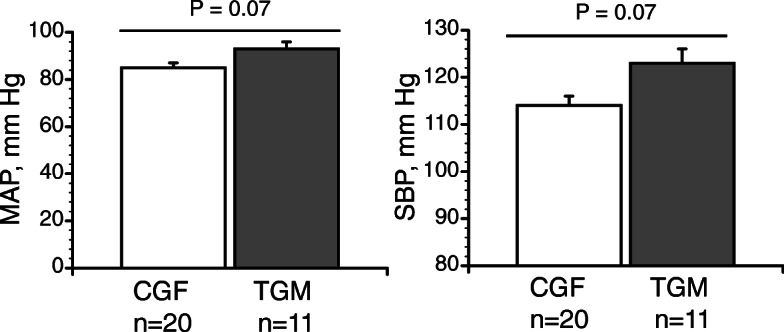
Trans men exhibit mild hypertension compared to cisgender females. Systolic blood pressure, SBP: 123 ± 3 vs. 114 ± 2 mm Hg, and mean blood pressure, MAP: 93 ± 3 vs. 85 ± 2 mm Hg (mean ± SEM). Blood pressure was taken supine by auscultation, following 15 min of rest. From Gulanski et al. [48], with permission

**Fig. 5:**
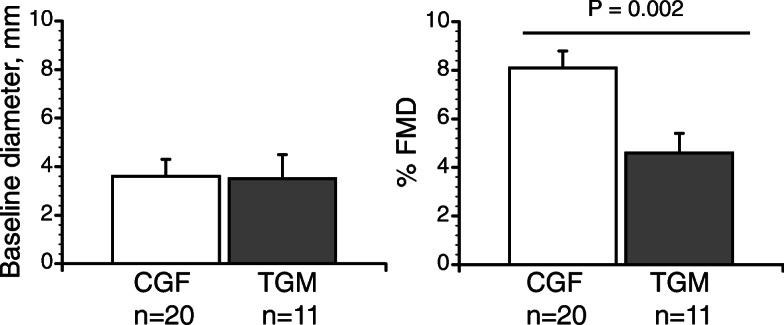
Trans men exhibit diminished endothelial function compared to cisgender females. Flow mediated vasodilation (%FMD): 4.5 ± 0.9 vs. 8.1 ± 1.0% (mean ± SEM). We used a standard protocol [76]. In each protocol, the subject lay supine, ultrasound brachial artery measures were recorded at baseline for 3 min. Forearm ischemia was then induced by rapidly inflating a blood pressure cuff to supra-systolic pressure (i.e., ≥ 160-180 mm Hg) for 5 min. Brachial artery measures were obtained continuously for 3 min following cuff deflation. FMD analysis is described in Gulanski et al. [48], with permission

In a recent study in our laboratory, systolic and mean blood pressures were slightly but not statistically higher in trans men (20–33 years) undergoing HT ([T_[Total]_ 196-1100 ng/mL) relative to cisgender females (18–36 years) (Fig. 4, *P* = 0.07) [48]. It is difficult to tell from these data whether these increases portend a risk of future organ damage or are a harbinger for future CAD [75] because DBP was not increased. Mild hypertension in trans men is not currently treated. Further study in these areas is crucial because even mild chronic elevations in blood pressure can result in organ damage, leading to frank hypertension. In this same study, we demonstrated markedly diminished endothelial function in trans men compared to cisgender women (Fig. 5) [48] and endothelial dysfunction is a key predictor of atherosclerosis. Again, we see an important sex difference because testosterone therapy decreases atherosclerotic CAD risk in older, hypogonadal cisgender men [77, 78], which has been attributed to improved or disrupted AR signaling pathways that govern lipid/lipoprotein metabolism [79].

Androgen administration in trans men is also associated with dyslipidemia [71, 80–82]. An observational study (45 months) compared lipid profiles in trans men receiving testosterone and trans men not taking HT [71]. Trans men taking HT had significantly less favorable lipid profiles, such that HDL-C levels were lower, total cholesterol was higher, and triglyceride levels were nearly double compared to controls. Similar results were reported in a separate observational study [80] and have also been confirmed in meta-analyses [81, 82].

While similar lipid abnormalities in women with AE-PCOS have also been demonstrated, these are more difficult to interpret and likely result from the combined effects of hyperandrogenism, obesity, and insulin resistance that are present with AE-PCOS. The interdependence of androgen and insulin contributions to lipid metabolism is recognized [79], but these effects can be independent in women with androgen excess [83], suggesting an important role for androgen exposure in the AE-PCOS women as well. That said, androgen control of lipid metabolism is not well understood in women and could also rely on estrogen action.

## Conclusions

Our data and others support an association between hyperandrogenism and mild elevations in blood pressure, endothelial dysfunction, and dyslipidemia in trans men and women with AE-PCOS. Despite the evidence that androgen exposure during gender-affirming hormone therapy is associated with mild hypertension, endothelial dysfunction, and dyslipidemia in trans men, the lack of long-term studies and infrequent follow-up measures for existing studies has led to uncertainty about the effects of HT on cardiovascular outcomes [80]. Cardiovascular markers are not always treated in trans men or AE-PCOS, leaving these cohorts at greater risk for cardiovascular events and future CAD, and even mild hypertension can be detrimental to the cardiovascular system when chronic. AE-PCOS is often diagnosed in the early teenage years, and gender transition often occurs at a young age. In both cases, androgen exposure will last for many years. Long-term and follow-up research are needed to develop guidelines for cardiovascular outcomes during HT and support health and longevity in trans men.

### Perspectives and significance

Despite the work presented in this review, the long-term health risks of testosterone exposure on the female vascular system and the impact of testosterone in women with AE-PCOS, and in trans men receiving androgens, remain underappreciated. In particular, attention to cardiovascular and metabolic risk factors should be integral to the care of these cohorts. The high, chronic androgen therapy environment in trans men provides a unique opportunity to study the impact of long-term androgen exposure on the female vascular system. This is especially important because the elevated androgens in trans men remain throughout their lifetime, continuing into older age when CAD risk develops independent of hormone exposure.

## Data Availability

N/A

## Abbreviations

Akt: Protein kinase B
AE-PCOS: Androgen excess-polycystic ovary syndrome
AR: Androgen receptor
BMI: Body mass index
CAD: Coronary artery disease
CVC %Max: Percent maximal cutaneous vasodilation
ERK: Extracellular signal-regulated kinases
ET-1: Endothelin-1
FMD: Flow-mediated vasodilation
GnRH ant: Gonadotropin-releasing hormone antagonist
GPR30 agonist G-1: G protein-coupled estrogen receptor 1
HDL-cholesterol: High-density lipoprotein cholesterol
HT: Hormone therapy
LDL-cholesterol: Low-density lipoprotein cholesterol
MAPK: Mitogen-activated protein kinase
MSNA: Muscle sympathetic neural activity
NF-κB: Nuclear factor kappa-light-chain-enhancer of activated B cells
NO: Nitric oxide
PCOS: Polycystic ovary syndrome
PI3K: Phosphatidylinositol 3-kinase
PKA/PKC: Protein kinase A/protein kinase C
SBP: Systolic blood pressure
TG: Triglycerides
Trans men: Female-to-male transgender individuals
